# Local Action of Increased Pressure Induces Hyperpolarization Electrical Signals and Influences Photosynthetic Light Reactions in Wheat Plants

**DOI:** 10.3390/plants12132570

**Published:** 2023-07-07

**Authors:** Lyubov Yudina, Alyona Popova, Yuriy Zolin, Ekaterina Sukhova, Vladimir Sukhov

**Affiliations:** Department of Biophysics, N.I. Lobachevsky State University of Nizhny Novgorod, 603022 Nizhny Novgorod, Russia; lyubovsurova@mail.ru (L.Y.); silverkumiho@mail.ru (A.P.); uchebnayap.zolin@gmail.com (Y.Z.); n.catherine@inbox.ru (E.S.)

**Keywords:** hydraulic waves, photosynthetic inactivation, quantum yield of photosystem II, nonphotochemical quenching, artificially increased pressure

## Abstract

Long-distance electrical signals caused by the local action of stressors influence numerous physiological processes in plants including photosynthesis and increase their tolerance to the action of adverse factors. Depolarization electrical signals were mainly investigated; however, we earlier showed that hyperpolarization electrical signals (HESs) can be caused by moderate stressors (e.g., local moderate heating) and induce photosynthetic inactivation. We hypothesized that HESs are related to stressor-induced increases in the hydrostatic pressure in the zone of action of the stressor and following the propagation of a hydraulic wave. In the current work, we tested this hypothesis through the direct investigation of electrical signals induced by the local action of artificially increased pressure and an analysis of the subsequent photosynthetic changes in the nonirritated parts of plants. The electrical signals and parameters of photosynthetic light reactions were investigated in wheat plants. The local action of the increased pressure was induced by the action of weights on the wheat leaf. Extracellular electrodes were used for electrical signal measurements. Pulse–amplitude–modulation fluorescent imaging was used for measurements of the quantum yield of photosystem II and nonphotochemical quenching of chlorophyll fluorescence in wheat leaves. It was shown that the local action of pressure on wheat leaf induced electrical signals near the irritated zone: HESs were caused by low pressure (10 kPa) and depolarization signals were induced by high pressure (100 kPa). The local action of moderate pressure (50 kPa) induced weak electrical signals near the irritated zone; however, HESs were observed with increasing distance from this zone. It was also shown that the local action of this moderate pressure induced the photosynthetic inactivation (decreasing the quantum yield of photosystem II and increasing the nonphotochemical quenching of chlorophyll fluorescence) in the nonirritated parts of the wheat leaves. Thus, our results show that the local action of the increased pressure and, probably, subsequent propagation of the hydraulic wave induce electrical signals (including HESs) and photosynthetic inactivation in nonirritated parts of plants that are similar to ones caused by the local action of moderate stressors (e.g., moderate heating). This means that both HESs and depolarization electrical signals can have a hydraulic mechanism of propagation.

## 1. Introduction

In environmental conditions, plants can often be influenced by adverse factors. The action of many factors (excess light, nonoptimal temperatures, drought, mechanical damages, etc.) can be spatially heterogenous. This means that plants should have long-distance stress signals providing a systemic adaptive response to the local action of adverse factors [1,2]. It is known that this action induces the propagation of long-distance electrical signals (ESs), which are transient changes in an electrical gradient across the plasma membranes of plant cells [1,2]. There are depolarization electrical signals (DESs), which include the initial depolarization of the plasma membrane and its subsequent hyperpolarization [3,4,5], and hyperpolarization electrical signals (HESs), which include the initial hyperpolarization and subsequent depolarization [6,7,8].

It is considered that DESs can increase a plant’s tolerance to the action of stressors [2,9], providing a systemic adaptive response [1]. This increase is probably based on numerous physiological changes that can be induced by DESs [1,9], including stimulation of the expression of defense genes (e.g., proteinase inhibitor genes [10] and the gene of vegetative storage protein 2 [11]), inactivation of photosynthesis (e.g., decreases in CO_2_ assimilation, quantum yield of photosystem I, quantum yield of photosystem II (Φ_PSII_), and linear electron flow and increases in the nonphotochemical quenching of chlorophyll fluorescence (NPQ) and cyclic electron flow around photosystem I) [1,2,12,13,14], activation of respiration [15,16,17], stimulation of the production of stress phytohormones (e.g., abscisic and jasmonic acids) [18,19,20], changes in the transpiration rate and stomata opening [21,22,23], and suppression of phloem mass flow [24,25,26], among others. The ways in which DES-induced specific physiological changes and increasing plant tolerance are related are actively being investigated [1,2,9]; particularly, stimulation of the cyclic electron flow around photosystem I and NPQ can decrease the photodamage of the photosynthetic machinery under the action of many stressors (e.g., excess light, nonoptimal temperatures, or drought) through the suppression of electron transport to O_2_ and reactive oxygen species production.

However, the participation of DESs in physiological regulation under environmental conditions can be limited. There are two main types of DESs in higher plants: action potentials and variation potentials [5,9]. Action potentials, which are short-term self-propagating spikes formed by the transient activation of Ca^2+^, anions, and outwardly rectifying K^+^ channels [27,28], as well as partly by the transient inactivation of H^+^-ATPase in the plasma membrane [29], can be induced by weakly intensive stimuli; however, a long-term refractory period is necessary for their induction. This means that action potentials can only propagate under favorable and stable conditions, which are rare in the environment [1].

In contrast, long-term and irregular variation potentials [3,30], caused by the transient activation of Ca^2+^ channels and the subsequent inactivation of H^+^-ATPase in the plasma membrane, can be induced by damaging stimuli (e.g., burning or strong heating) under both favorable and stress conditions (e.g., under a water deficit [31]). This means that variation potentials can be induced under extreme situations inducing these damaging stimuli (e.g., wildfire), i.e., these signals are also not typical under environmental conditions [8,32].

Our previous works [8,32] showed that HESs, which are classified as system potentials [6,7], can also participate in forming systemic physiological responses in plants on the local action of environmental factors, because these signals can be induced by moderate stressors (e.g., local heating to 40 °C) and inactivate photosynthetic processes in nonirritated parts of plants (decrease Φ_PSII_ and increase NPQ). These photosynthetic changes are in a good accordance with the changes induced by DESs [12,13,14,31] and show principal similarity between DES- and HES-induced photosynthetic responses. As a result, an analysis of the mechanisms of the generation and propagation of HESs can be important for understanding their influence on physiological processes.

In accordance with Zimmermann et al. [6], the activation of H^+^-ATPase in the plasma membrane is considered the main mechanism of HESs (system potentials), because the generation of these signals is strongly dependent on the initial activity of the H^+^-ATPase and can be imitated by action of the activator of this transporter. However, the mechanisms of HESs’ propagation are not clear; interactions among H^+^-ATPases in the plasma membrane or the propagation of a wave of a decreased Ca^2+^ concentration in the apoplast were proposed as potential mechanisms of this propagation [1,6].

Earlier, we hypothesized that HESs can be caused by the propagation of a hydraulic wave with low magnitudes from the irritated zone [8,32]. There are several arguments supporting this hypothesis. First, HESs can be formed from DESs (variation potentials) with increasing distance from the irritated zone [8]. Variation potentials are considered to be caused by the propagation of hydraulic waves and the subsequent activation of mechanosensitive Ca^2+^-channels inducing the Ca^2+^ influx and H^+^-ATPase inactivation [30,33,34,35]. The magnitudes of these waves decrease with increasing distance from the irritated zone [34,36], i.e., this decrease can explain the transformation of variation potentials into HESs.

Second, local burning induces DESs (variation potentials) under irrigation or a moderate water deficit; however, this burning can induce HESs under a strong water deficit that is accompanied by a decreasing relative water content in plants [31]. This strong water deficit should decrease hydrostatic pressure changes under the local action of stressors; i.e., in this case, the transformation of DESs into HESs is also related to the decreasing magnitude of the hydraulic wave.

Third, some works show the stimulation of H^+^-ATPase in the plasma membrane via increased hydrostatic pressure [37]. This stimulation can be mediated by the weak activation of mechanosensitive Ca^2+^ channels and a weak increasing calcium concentration in the cytoplasm [8,32], because this increase can induce the absolute activation of H^+^-ATPase [38,39,40] or relative activation of this transporter in comparison to inwardly rectifying K^+^ channels through the Ca^2+^-dependent inactivation of the channels [41,42]. It should be noted the strong activation of mechanosensitive Ca^2+^ channels and large increasing Ca^2+^ concentration in the cytoplasm that accompany variation potentials, caused by the inactivation of H^+^-ATPase in the plasma membrane [1,29].

Thus, the aim of the current work was to test a hypothesis concerning the participation of hydraulic waves in the induction of HESs. To check this hypothesis, we analyzed ESs induced by the local action of artificially increased pressure on the wheat leaf and investigated subsequent changes in Φ_PSII_, and NPQ in nonirritated parts of this leaf. This pressure action should directly induce the propagation of a hydraulic wave through a wheat leaf and can be considered as an experimental model for the analysis of the hydraulic hypothesis of HESs’ propagation. The similarity of the electrical signals and photosynthetic changes induced by the local pressure action with the signals and changes induced by the local action of stressors (e.g., the moderate heating), which has previously been shown [31,32], should support this hypothesis; in contrast, the absence of this similarity should be an argument against this proposition.

## 2. Results

### 2.1. Induction of Electrical Signals by the Local Action of Increased Pressure

**Figure 1:**
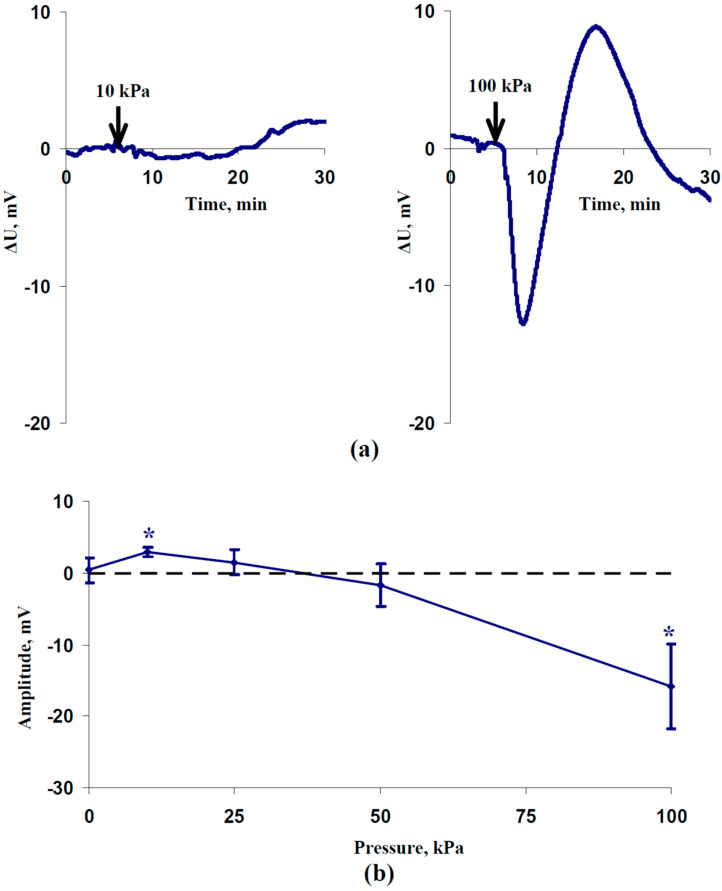
Records of the (**a**) hyperpolarization electrical signal (HES) and depolarization electrical signal (DES) induced by 10 and 100 kPa pressures, respectively; (**b**) dependence of the average amplitude of the electrical signals on the magnitude of the increase in pressure (*n* = 9–10) 0 cm from the irritated zone. ΔU is the difference in the electrical potentials between the measuring and reference electrodes. Positive values of ΔU show hyperpolarization; negative values show depolarization. It is also assumed that HESs have positive amplitudes and DESs have negative amplitudes. * Average amplitudes (blue solid line with markers) are significant in comparison to zero (black dotted line) (*p* < 0.05).

An analysis of ESs induced by the local action of increased pressure shows that the type of electrical signal was strongly dependent on the pressure’s magnitude. The local action of 10 kPa of pressure induced small and slow hyperpolarization at a 0 cm distance from the irritated zone (Figure 1a); this response can be classified as an HES [8]. In contrast, 100 kPa of pressure caused a DES that included fast depolarization, followed by repolarization, and, finally, slow depolarization. The dependence of the average amplitude of ESs on the pressure’s magnitude included two phases (Figure 1b): an increase in the amplitude of HESs with a weak increase in the hydrostatic pressure and an increase in the amplitude of DESs with a strong increase in this pressure.

This result supports the possibility of the induction of both HESs and DESs under the different magnitudes of the hydraulic wave, which was hypothesized in our previous works [8,32].

**Figure 2:**
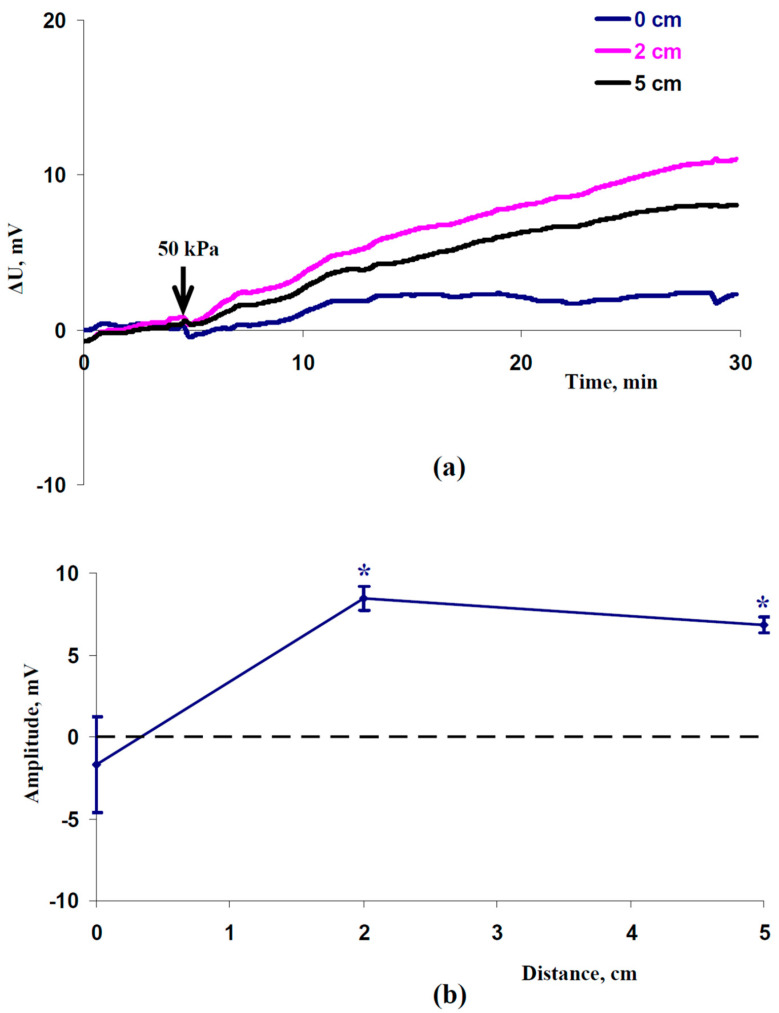
Records of the (**a**) hyperpolarization electrical signals induced by 50 kPa of pressure and (**b**) dependence of the average amplitude of these signals on the distance from the irritated zone (*n* = 9). ESs were measured at 0, 2, and 5 cm from the irritated zone. ΔU is the difference in the electrical potentials between the measuring and reference electrodes. Positive values of ΔU show hyperpolarization; negative values show depolarization. It is also assumed that HESs have positive amplitudes and DESs have negative amplitudes. * Average amplitudes (blue solid line with markers) were significant in comparison to zero (black dotted line) (*p* < 0.05).

Considering a decrease in the magnitude of the hydraulic wave with an increase in the distance from the irritated zone [34,36], we supposed that the type of ES should change with a change in this distance. It was shown that only a weak ES was observed at a 0 cm distance from the irritated zone; in contrast, HESs were generated at 2 and 5 cm distances (Figure 2a).

An analysis of the dependence of the average amplitudes on the distance from the irritated zone supported the absence of significant amplitudes of electrical signals at a 0 cm distance and the appearance of significant amplitudes of hyperpolarization signals at 2 and 5 cm distances (Figure 2b). It should be noted that the average amplitudes of the HESs were approximately 8.5 mV (2 cm) and 7 mV (5 cm). These amplitudes were similar to the amplitudes of the HESs induced by the local action of the moderate heating or a combination of this heating and illumination [8,32].

Our results show that the local action of increased pressure could induce HESs that were similar to HESs induced by the action of moderate stressors; this similarity supports the hydraulic mechanism of the hyperpolarization electrical signals [8]. However, we earlier showed that HESs decreased the Φ_PSII_ and increased NPQ [32]; these results were in good accordance with the HES-induced photosynthetic inactivation shown by other authors [23,43]. Thus, we analyzed the influence of the local action of the increased pressure on the photosynthetic parameters in the next stage of the investigation.

### 2.2. Induction of Photosynthetic Changes by the Local Action of Increased Pressure

**Figure 3:**
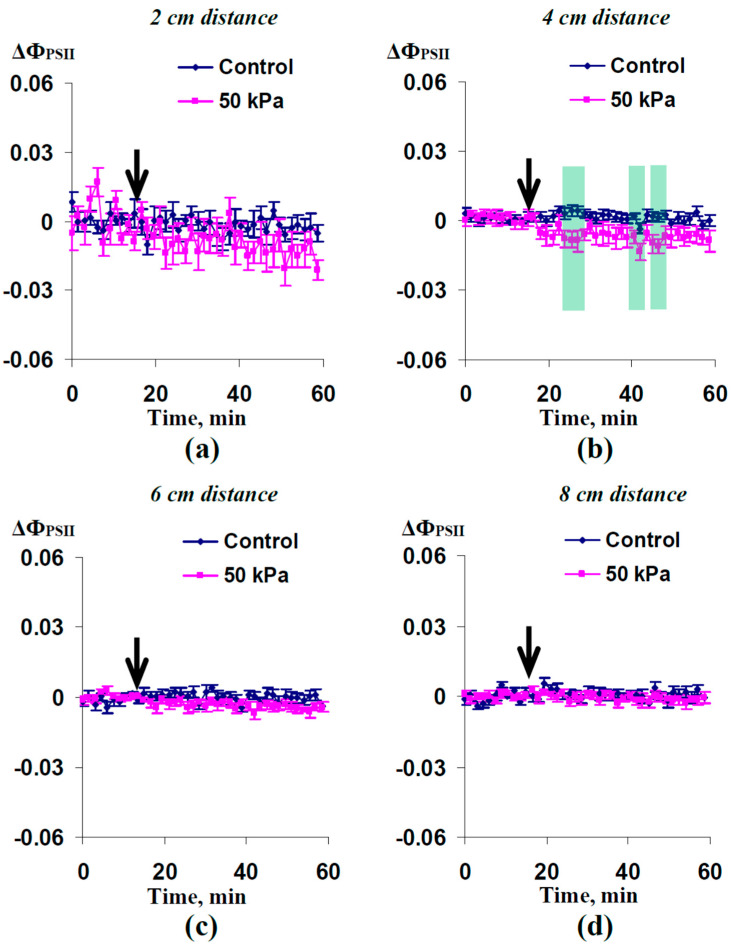
Averaged changes in the quantum yield of photosystem II (ΔΦ_PSII_) at (**a**) 2 cm, (**b**) 4 cm, (**c**) 6 cm, and (**d**) 8 cm from the zone of local action of 50 kPa of pressure (*n* = 14). The arrows mark the initiation of this action; control plants were not irritated. ΔΦ_PSII_ was calculated as Φ_PSII_ − Φ_PSII_^0^, where Φ_PSII_^0^ was measured before the initiation of the pressure action. The green shading shows significant differences between the experimental and control values of ΔΦ_PSII_ (*p* < 0.05).

It was shown (Figure 3) that the local action of the 50 kPa pressure and, probably, HESs induced by this pressure caused a weak decrease in the quantum yield of photosystem II at 4 cm from the irritated zone, which was significant in several time ranges. Despite the small magnitude of the effect, it was similar to the decrease in Φ_PSII_, which was observed in nonirritated parts of the plant after the local action of the moderate heating or a combination of this heating and illumination [32]. Significant changes at other distances from the irritated zone were absent (Figure 3a,c,d).

**Figure 4:**
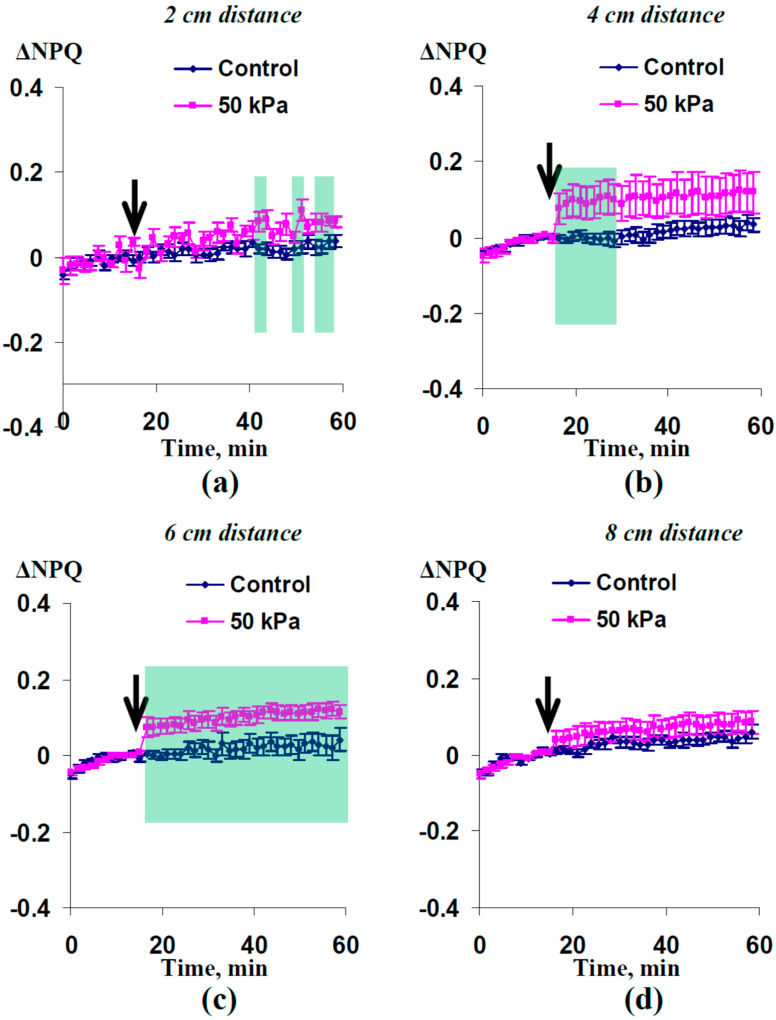
Averaged changes in the nonphotochemical quenching of the chlorophyll fluorescence (ΔNPQ) at (**a**) 2 cm, (**b**) 4 cm, (**c**) 6 cm, and (**d**) 8 cm from the zone of local action of the 50 kPa pressure (*n* = 14). The arrows mark the initiation of this action; control plants were not irritated. ΔNPQ was calculated as NPQ − NPQ^0^, where NPQ^0^ was measured before the initiation of the pressure action. The green shading shows significant differences between the experimental and control values of ΔNPQ (*p* < 0.05).

The nonphotochemical quenching of the chlorophyll fluorescence was also influenced by the local action of the 50 kPa pressure and HESs (Figure 4). A significant increase in NPQ was observed at 2, 4, and 6 cm distances from the irritated zone; significant changes in NPQ were absent at a 8 cm distance. It should be noted that the increases in NPQ were more expressive than the decreases in Φ_PSII_; the size of the zone with changes in the nonphotochemical quenching was larger than the size of the zone with changes in the quantum yield of photosystem II. These characteristics were similar to the characteristics of the photosynthetic response induced by the local action of the moderate heating or a combination of this heating and illumination [32].

Thus, the photosynthetic investigation also showed similarity between the effects induced by the local action of the increased pressure and the effects caused by the local action of moderate stressors (heating, heating and illumination). These results additionally support hydraulic mechanisms of HESs, which were earlier proposed [8,32].

## 3. Discussion

ESs are a probable important mechanism of the induction of a fast systemic adaptive response in plants under the local action of stressors [1,2,9]. The participation of ESs and ES-induced physiological changes in the formation of this response is traditionally investigated through the analysis of DESs, including action potentials and variation potentials [3,4,5]. However, propagation of action potentials in higher plants requires favorable and stable conditions [1]; variation potentials, rather, are “emergency” electrical signals that can be induced by rare and extreme stressors [8,32]. Thus, both DESs cannot be a common phenomenon under environmental conditions.

Earlier, we showed [8,32] that HESs can be induced by the local action of moderate stressors (e.g., heating to 40 °C) and influence photosynthetic processes, decreasing Φ_PSII_ and increasing NPQ; these photosynthetic changes are similar to the DES-induced response of photosynthesis [12,13,14,31]. The results show that HESs (particularly, system potentials [6,7]) can play key role in the induction of the fast systemic adaptive response in higher plants, because they can be caused under environmental conditions. This means that the HESs require extensive investigations, including analyses of their influence on plant tolerance to stressors, studies of inductions of HESs in plants of different species and/or under different conditions, investigations of the mechanisms of these hyperpolarization signals and their influence on physiological process, etc.

Our current work focused on the analysis of the hydraulic hypothesis of mechanisms of HESs. In accordance with this hypothesis [8,31,32], hydraulic waves with low magnitudes can induce the absolute or relative activation of H^+^-ATPase in the plasma membrane. This hypothesis is in good accordance with the hypothesis concerning the induction of variation potentials by hydraulic waves with high magnitudes [30,33,34,35], because variation potentials can be transformed into DESs at propagation [6,7,8,31].

The results of the current work support the hydraulic hypothesis through several important points. First, the dependence of the amplitude of ES on the magnitude of the increased pressure shows a two-phase response (Figure 1): induction of HESs under the local action of low pressure (10 kPa) and induction of DESs under the local action of high pressure (100 kPa). This result can explain the induction of HESs (probably, system potentials) under the local action of moderate stressors [8,32] and induction of DESs (variation potentials) under the local action of strong stressors [12,13,14,31], because the magnitude of the increased hydrostatic pressure should be dependent on the intensity of the stressor action.

Second, transformation of weak ESs into HESs with increasing distance from the zone of the 50 kPa pressure action (Figure 2) is in good accordance with the similar transformation of variation potentials into HESs under the local action of burning, moderate heating, and a combination of heating and illumination [8,31,32]. This effect is probably related to a decrease in the magnitude of the hydrostatic pressure in the xylem with an increase in the distance from the zone of action of the increased pressure or stressor [34,36].

Third, the local action of the moderate hydrostatic pressure (50 kPa) decreases Φ_PSII_ (Figure 3) and increases NPQ (Figure 4). These responses are similar to the photosynthetic responses induced by both HESs [8,23,31,32,43] and DESs [12,13,14,16] under the local action of stressors of different types (burning, strong and moderate heating, re-irrigation, and others). It is probable that the mechanisms of HES- and DES-induced photosynthetic activation are similar, because both signals are accompanied by apoplast alkalization [6,12], which participates in ES-induced photosynthetic inactivation [1,9,44]. Considering the participation of the DES-induced photosynthetic response in the increase in plant tolerance to the action of stressors [1,9], it can be proposed that HES-induced photosynthetic changes also increases this tolerance.

Thus, our results support the hydraulic hypothesis of the propagation of HESs. This hypothesis is also in good accordance with data in the literature. It is known that increased hydrostatic pressure can both activate and suppress H^+^-ATPase in the plasma membrane under different experimental conditions [34,37]. It is probable that the influence of the hydraulic signal can be related to the activation of mechanosensitive Ca^2+^ channels and an increasing concentration of calcium ions in the cytoplasm [1]. Potentially, a weak increase in the Ca^2+^ concentration can induce the activation of H^+^-ATPase [38,39,40] and/or inactivation of inwardly rectifying K^+^ channels [41,42]; the last way can provide a relative increase in the activity of H^+^-ATPase in comparison to potassium ion channels. In contrast, a strong increase in the Ca^2+^ concentration should suppress H^+^-ATPase and induce DESs.

Finally, our results also show that HESs are potentially induced under moderate local mechanical impacts on plants, because these impacts can be accompanied by pressing of the leaf. Our work was not focused on the analysis of this possibility because the long-term action of increased pressure was only investigated. However, an analysis of ESs and photosynthetic changes induced by the short-term local action of increased pressure can be important problem for future investigations, because the short-term mechanical impact on parts of the plant seems to be more probable under environmental conditions than a long-term one. Additionally, an analysis of ESs and photosynthetic changes induced by the local action of increased pressure on different areas of this action can be also important for future investigations of the role of HESs under moderate local mechanical impacts on parts of plants.

## 4. Materials and Methods

### 4.1. Plant Material and Local Action of Increased Pressure

Spring wheat plants (*Triticum aestivum* L., cultivar “Daria”) were cultivated for 13–14 days in the vegetation room of the Department of Biophysics (N.I. Lobachevsky State University of Nizhny Novgorod, Nizhny Novgorod, Russia) in accordance with our previous works [8,32]. After 2 days of soaking, the wheat seedlings were planted in pots with universal soil; there were two plants per pot. Wheat plants were cultivated under a 16 h light period and 8 h dark period. A 24 °C temperature was provided for both the dark and light periods of the wheat cultivation. The luminescent lamps FSL YZ18RR (Foshan Electrical And Lighting Co., Ltd., Foshan, China) were used as the source of white light with the intensity equaling approximately 100 μmol m^−2^ s^−1^. The plants were irrigated three times a week.

**Figure 5:**
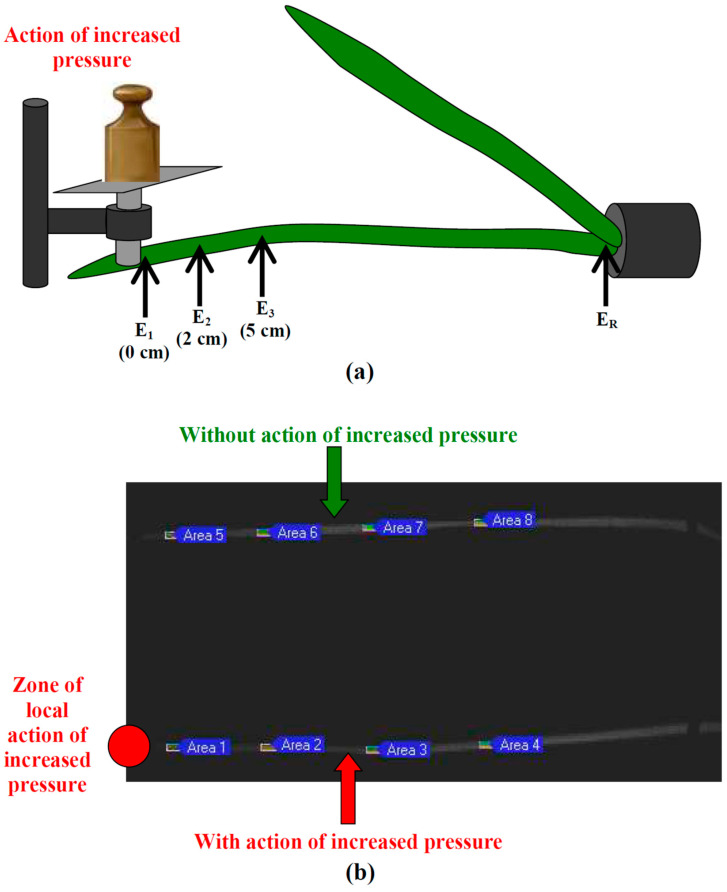
(**a**) Scheme of the localization extracellular electrodes and system providing the local action of the increased pressure on wheat leaf. E_1_, E_2_, and E_3_ are the measuring electrodes; E_R_ is the reference electrode. The 20, 50, 100, and 200 g weights were used for the induction of the local action of the 10, 25, 50, and 100 kPa pressures in the electrophysiological measurements. (**b**) Example of localizations of ROIs in experimental and control wheat plants. The 100 g weight was used for the induction of the local action of the 50 kPa pressure in the photosynthetic measurements.

The local action of the artificially increased pressure on the tip of the second wheat leaf was provided by a self-manufactured system (Figure 5a), which included a glass piston with a platform, a tube for piston positioning, and a series of weights. The glass piston was superimposed on the wheat leaf before the initiation of experiment. Its diameter was 5 mm; this diameter was smaller than the width of the investigated wheat leaves in the irritated zone. This was necessary for the elimination of the influence of this width on the pressure provided by the glass piston. The local action of pressure (initiation of irritation) was induced by the superimposition of the weight on the platform after 60 min of adaptation; the falling of this weight on the platform during superimposition was excluded. This procedure provided step increases of the pressure. The superimposed weight was not changed for the remaining experimental time. Different weights including 20, 50, 100, and 200 g were used and provided 10, 25, 50, and 100 kPa pressures, respectively. The weight was not superimposed in the control; this variant was assumed as the 0 kPa pressure.

### 4.2. Electrophysiological Measurements

In accordance with our previous works [8,32], extracellular Ag^+^/AgCl electrodes (RUE Gomel Measuring Equipment Plant, Gomel, Belarus), a high-impedance IPL-113 amplifier (Semico, Novosibirsk, Russia), and a personal computer were used for the extracellular measurements of the ESs. The ESs were measured in the second mature leaf of the wheat plants. The measuring electrodes were placed 0 cm from the border of the irritated zone (zone of action of the increased pressure) for experiments with 10, 25, and 100 kPa pressures and 0, 2, and 5 cm from this zone for experiments with 50 kPa pressure. The reference electrode was contacted to the wheat stem near the soil. These electrodes were contacted to the plant via Uniagel conductive gel (Geltek-Medica, Moscow, Russia).

After the placement of the electrodes, wheat plants were adapted for 60 min before the initiation of the local action of the increased pressure; the duration of measurement after this local irritation was approximately 25 min.

### 4.3. Measurements of Parameters of Photosynthetic Light Reactions

The quantum yield of photosystem II (Φ_PSII_) and nonphotochemical quenching of chlorophyll fluorescence (NPQ) were measured with using a PAM imaging Open FluorCam FC 800-O/1010 (Photon Systems Instruments, Drasov, Czech Republic) in accordance with our previous work [32].

The first standard saturation pulse of this system (4000 μmol m^−2^ s^−1^, cold white light, 6500 K) after 15 min dark adaptation was used for the estimation of the maximum fluorescence (Fm) [45]. The next saturation pulses were used for the calculation of the maximum fluorescence in the light (Fm’) and steady-state value of fluorescence immediately prior to the saturation pulse (Ft). These pulses were generated every 90 s after 30 min illumination with white actinic light (456 μmol m^−2^ s^−1^, cold white light, 6500 K); this illumination was not changed for the remaining experimental time (60 min). The duration of the photosynthetic measurements (duration of the repeating generation of saturation pulses) before the initiation of the local action of the increased pressure was 15 min; this duration after the initiation was 45 min. The 50 kPa pressure was used in the photosynthetic experiments. Φ_PSII_ and NPQ were calculated on the basis of Fm, Fm’, and F in accordance with standard equations [45] for each saturation pulse.

Two wheat plants (the irritated experimental plant and nonirritated control plant) were simultaneously investigated in each repetition in the experiments with photosynthetic measurements (Figure 5b). The photosynthetic parameters were analyzed in four ROIs with the same length (approximately 0.5 cm) in each wheat plant (the second mature leaf) with centers located 2, 4, 6, and 8 cm from border of the irritated zone.

In accordance with Yudina et al. [32], ΔΦ_PSII_ and ΔNPQ were used in the investigation. ΔΦ_PSII_ was calculated as Φ_PSII_ − Φ_PSII_^0^, and ΔNPQ was calculated as NPQ − NPQ^0^, where Φ_PSII_^0^ and NPQ^0^ were Φ_PSII_ and NPQ before the initiation of the local action of pressure. Φ_PSII_^0^ and NPQ^0^ were calculated as the averaged Φ_PSII_ and NPQ for three measuring points before the initiation of the pressure action. Using ΔΦ_PSII_ and ΔNPQ decreased the individual variability of the photosynthetic parameters in the investigated wheat plants.

### 4.4. Statistics

Each experimental or control group included from 9 to 14 separate plants; these quantities are shown in the caption of the figures. The representative records, averaged records, mean values, and standard errors are presented in the figures. The Student’s *t*-test was used for the estimation of the significance.

## 5. Conclusions

In the current work, we showed that the local action of increased pressure could induce both hyperpolarization electrical signals (10 kPa pressure) and depolarization electrical signals (100 kPa pressure) in wheat plants. The type of signal was also dependent on the distance from the irritated zone, because the local action of the 50 kPa induced weak electrical signals at the 0 cm distance and hyperpolarization electrical signals at the 2 and 5 cm distances. An analysis of the parameters of the photosynthetic light reactions showed that the local action of the 50 kPa pressure decreased Φ_PSII_ and increased NPQ in the nonirritated parts of leaves. Changes in Φ_PSII_ were observed at the 4 cm distance from the zone of the increased pressure action; changes in NPQ were observed at the 2–6 cm distance.

Thus, the local action of the increased pressure, which should directly cause the propagation of the hydraulic wave through the wheat leaf, induced different types of electrical signals (hyperpolarization and depolarization signals), depending on the pressure’s magnitude and distance from the irritated zone, and suppressed photosynthetic processes in the nonirritated parts of the plant. The observed responses were strongly similar to the electrical signals and photosynthetic changes under the local action of moderate stressors in our previous works (e.g., moderate heating [31,32]). These results support the hydraulic hypothesis of the propagation of hyperpolarization electrical signals in plants and show that a hydraulic wave with low magnitude can induce hyperpolarization electrical signals; in contrast, a hydraulic wave with high magnitude induces depolarization electrical signals.

## Data Availability

The data presented in this study are available upon request from the corresponding author.
